# Random Vibration Evaluation and Optimization of a Flexible Positioning Platform Considering Power Spectral Density

**DOI:** 10.3390/s26020514

**Published:** 2026-01-13

**Authors:** Lufan Zhang, Mengyuan Hu, Heng Yan, Hehe Sun, Zhenghui Zhang, Peijuan Wu

**Affiliations:** School of Mechanical and Electrical Engineering, Henan University of Technology, 100 Lianhua Street, Zhengzhou 450001, China; hu17638043296@gmail.com (M.H.); yh17513271182@163.com (H.Y.); 13223903302@163.com (H.S.); 16638742191@163.com (Z.Z.); 18623714220@163.com (P.W.)

**Keywords:** ultra-high acceleration macro–micro motion platform, flexible positioning platform, random vibration, power spectral density, fatigue life

## Abstract

The flexible positioning platform is a critical structural component in the ultra-high acceleration macro–micro motion platform, enabling precise positioning across multiple scales. However, under high-frequency start–stop cycles and prolonged multi-condition operation, it is prone to fatigue damage induced by random vibrations, which poses a threat to system reliability. This study proposes a method for evaluating and optimizing the platform’s performance under random vibration based on power spectral density (PSD) analysis. In accordance with the IEC 60068-2-64 standard, representative load spectra from Tables A.8 and A.6 were selected as excitation inputs. Frequency-domain analyses of stress, strain, and displacement were conducted using ANSYS Workbench 2022R1 in conjunction with the nCode platform, incorporating the Gaussian three-sigma probability interval. The results reveal that stress and deformation are highly concentrated in the hinge region, indicating a structural vulnerability. Fatigue life predictions were carried out using the Dirlik method and Miner’s linear damage rule under various PSD loading conditions. The findings demonstrate that hinge stiffness is a key factor influencing vibration resistance and service life. This research provides theoretical support for the design optimization of flexible structures operating in complex random vibration environments.

## 1. Introduction

With the widespread application of ultra-high-acceleration macro–micro motion platforms in fields such as precision manufacturing, aerospace, and micro-nano positioning, increasing attention has been paid to issues of structural safety and reliability. As a core component responsible for both large-scale movement and micro-scale precision regulation, the flexible positioning platform is particularly vulnerable to multi-frequency random vibrations during the platform’s start–stop processes [1,2]. The compliant positioning platform is a precision mechanism that achieves motion through the elastic deformation of flexure hinges, allowing for smooth, continuous, and backlash-free displacement. Unlike traditional mechanical joints, it relies on the material’s elasticity, eliminating friction and wear, which makes it ideal for micro- and nano-scale applications requiring high accuracy and repeatability. These platforms are widely used in fields such as optical alignment, micro-manipulation, and semiconductor manufacturing, and they offer advantages in compactness, mechanical simplicity, and dynamic responsiveness. Under prolonged operation, random loads may induce structural fatigue failure, severely compromising the dynamic performance and service life of the entire system [3]. Traditional structural analysis methods primarily focus on deterministic static or harmonic loads, making it difficult to comprehensively capture the structural responses and fatigue risks under random excitation. Power spectral density (PSD), which characterizes the energy distribution of random signals in the frequency domain, has emerged as a key tool for random vibration evaluation [4,5,6,7,8,9]. Both domestic and international researchers have conducted extensive studies on random vibration-induced fatigue using PSD in areas such as electronic components, aerospace equipment, and automotive parts. However, systematic investigations of flexible mechanisms—particularly flexible positioning platforms—remain limited [10,11,12,13].

To address these gaps, this paper presents a PSD-based study on the random vibration response and fatigue life prediction of a flexible positioning platform [14,15,16]. Following the IEC 60068-2-64 standard, typical PSD profiles from Tables A.8 and A.6 are selected to simulate representative operating conditions [17]. IEC 60068-2-64 is an internationally recognized standard for environmental testing that specifies methods to assess the structural response of products subjected to broad-band random vibrations. It provides guidance for selecting appropriate power spectral density (PSD) excitation profiles that replicate realistic mechanical environments during transport or operation. Specifically, Table A.6 outlines a test profile for high-intensity vibration conditions, typically representing heavy-duty transport environments, while Table A.8 corresponds to a general vibration spectrum applicable to lighter equipment or standard shipping conditions. In this study, standardized datasets are utilized to define input power spectral density (PSD) curves for simulation, thereby ensuring the loading conditions accurately represent operational scenarios encountered by compliant positioning stages. Future work will investigate high-frequency operational characteristics under driving modes employing series-wound voice coil motors. A frequency-domain response analysis is conducted using the modal superposition method in ANSYS Workbench, while fatigue damage is evaluated using the Dirlik method and Miner’s rule within the nCode platform [17,18,19]. This analysis has identified the key stress and deformation areas, especially around the bending hinges, and proposed structural optimization strategies to enhance the vibration resistance and durability. This work provides a theoretical basis and practical guidance for the design and optimization of flexible structures in complex and highly vibrational service environments.

In recent years, a growing body of literature has explored random vibration fatigue using power spectral density (PSD)-based approaches across various engineering domains. For example, Zhang et al. [4] and Mi et al. [5] applied multi-axial fatigue evaluation frameworks to assess components subjected to stochastic loads. Wu et al. [12] proposed improved probabilistic models for stress amplitude prediction under PSD excitation, while Gao et al. [13] investigated the dynamic behavior of functionally graded structures under broadband vibration. Despite this progress, most studies have focused on rigid-body systems such as automotive structures or aerospace components. Research targeting compliant mechanisms—particularly those relying on elastic deformation for precision actuation—remains scarce [7,8]. Moreover, existing fatigue evaluations often neglect standardized loading environments such as those defined in IEC 60068-2-64 [14,15,16], which limits their applicability in real-world transport or operation scenarios.

This paper studies the influence of vibration factors caused by the ultra-high acceleration macro–micro motion platform under working conditions and proposes a method of random excitation vibration based on power spectral density applied to the platform. The present study addresses these gaps by systematically integrating IEC-standardized PSD profiles with finite element modal analysis and fatigue life prediction based on Dirlik’s method. The research provides a comprehensive evaluation of vibration-induced fatigue in compliant positioning platforms and proposes targeted design optimizations. Compared to existing works, this study offers a more realistic simulation framework and contributes to the fatigue-resilient design of precision flexure mechanisms.

## 2. Random Vibration Analysis of the Flexible Positioning Platform

The ultra-high acceleration macro–micro motion platform achieves precise positioning through multi-condition reciprocating start-stop operations, leading to structural fatigue over long-term use. The compliant positioning platform is the core structure for precise positioning in this platform. The connecting arm has a higher natural frequency, so vibration has a minimal impact on it. This paper focuses on the random vibration analysis and random vibration fatigue analysis of the compliant positioning platform [20,21,22]. The vibration study is conducted with Power Spectral Density (PSD) as the excitation condition. Based on the IEC 60068-2-64 standard, the random vibration analysis evaluates the corresponding stress and strain contours using the Gaussian distribution three-sigma range probability method. Using ANSYS Workbench in conjunction with nCode, random vibration fatigue predictions are made to study the vibration characteristics of the compliant positioning platform under different PSD loads. The Dirlik method and Miner’s cumulative damage theory are employed to predict the fatigue life due to random vibrations [23].

### 2.1. Principle of Ultra-High Acceleration Macro–Micro Motion Platform

**Figure 1:**
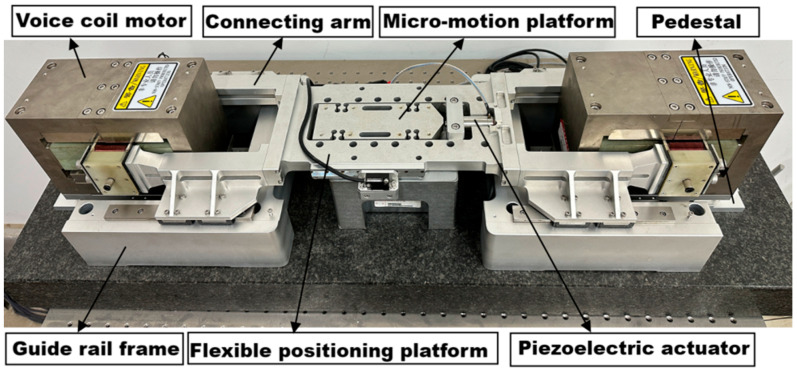
Ultra-high acceleration macro–micro motion platform.

The ultra-high acceleration macro–micro motion platform combines macro-micro positioning technology and integrates macro and micro drive systems to achieve high acceleration, high-precision control, and nanometer-level positioning accuracy [24,25,26,27,28,29]. The main components constituting the ultra-high acceleration macro-micro motion platform include piezoelectric actuators, flexible positioning platforms, guide rails, voice coil motors, connecting arms, grating rulers, and other components. The ultra-high acceleration macro–micro motion platform is shown in Figure 1. The voice coil motor and the piezoelectric actuator are the main driving units. The double voice coil motors are located at both ends of the platform to complete large-stroke macro positioning. The piezoelectric actuator provides precise compensation to ensure micro-positioning accuracy. Four actuating connecting arms connect the voice coil motor and the flexible platform to transmit motion loads. The platform realizes stable operation and high-precision positioning under ultra-high acceleration conditions. A guide rail is set under the flexible platform, and measurement devices such as grating rulers are used to improve stability and measurement accuracy. The base supports the entire platform structure and is fixed on the marble baseplate. The flexible positioning platform is a key core component, which bears the macro–micro drive effect and determines the overall accuracy of the system. The design and performance optimization of key components to achieve high acceleration and high precision performance of the platform have high application value and research significance for its multiple complex analyses.

The ultra-high-acceleration macro–micro motion platform operates under the following five specific positioning conditions:

1. Combined Drive: The voice coil motor and piezoelectric actuator work simultaneously to drive the platform for precise positioning.

2. Synchronous Forward Drive: Two sets of voice coil drive units move forward in unison to enable coordinated forward motion of the system.

3. Piezoelectric-Only Forward Drive: Only the piezoelectric actuator is activated for forward movement, while the voice coil motors remain stationary.

4. Synchronous Reverse Drive: The two voice coil motors operate synchronously in reverse, with the piezoelectric actuator remaining inactive.

5. Piezoelectric-Only Reverse Drive: Only the piezoelectric actuator performs reverse displacement, while the voice coil motor system does not contribute to the motion.

### 2.2. Finite Element Modal Analysis

A three-dimensional finite element model of the compliant positioning platform was established using ANSYS Workbench. The model includes critical structural components such as the compliant flexure hinges, connecting arms, and base. The mesh was locally refined in the flexure hinge region to improve the accuracy of stress response calculations. Material properties were assigned based on actual engineering parameters. Prior to the random vibration analysis, a modal analysis was conducted to extract the first six natural frequencies. These results, summarized in Table 1, served as the basis for the subsequent frequency-domain analysis using the modal superposition method [30,31,32].

### 2.3. Random Vibration Analysis Based on Table A.8

**Figure 2:**
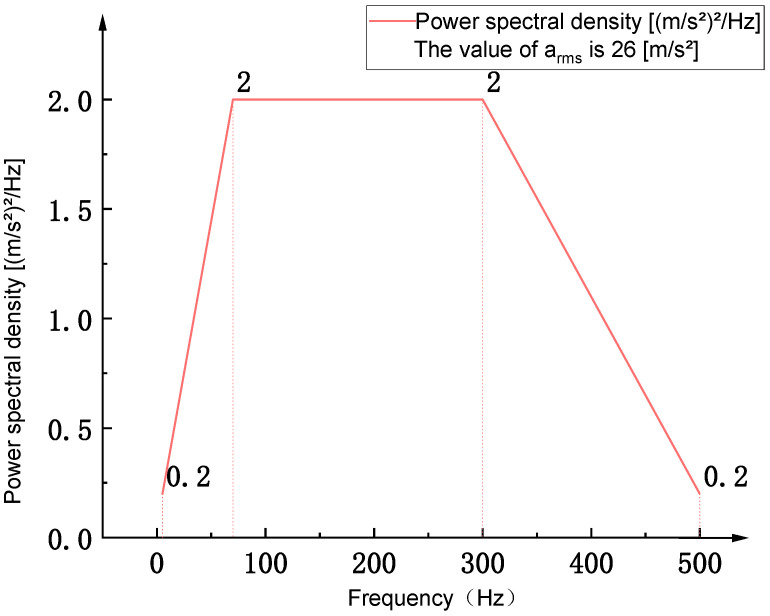
Table A.8 standard acceleration power spectral density curve.

The Power Spectral Density (PSD) describes the distribution characteristics of random vibration energy in the frequency domain and serves as a critical basis for analyzing structural vibration and assessing fatigue performance. PSD quantifies the energy distribution per unit frequency bandwidth. Frequency-domain analysis using PSD enables effective evaluation of the flexible positioning platform’s response to different frequency bands, thereby facilitating structural optimization. In this study, the selection of 0–500 Hz as the input frequency band for the power spectral density is based on the working frequency range of the voice coil motor (VCM), which is mainly excited by the flexible positioning platform. The PSD loading spectrum is defined based on Table A.8 of the IEC 60068-2-64:2019 standard to evaluate the structural safety of the flexible positioning platform under a random vibration environment with a root mean square (RMS) acceleration of 26 m/s^2^. As shown in Figure 2, a representative PSD loading curve is presented, covering a frequency range of 0–500 Hz. The curve exhibits a trapezoidal shape, with the Acceleration Spectral Density (ASD) maintaining a stable value of 2 (m/s^2^)^2^/Hz in the 70–300 Hz range, while gradually tapering to 0.2 (m/s^2^)^2^/Hz at both ends of the spectrum. Given the increasing research focus on flexible positioning platforms operating in the 2g acceleration region, a PSD curve with an RMS acceleration of 26 m/s^2^ is selected to assess the overall vibration intensity characteristics under random excitation. Based on the available experimental conditions, this PSD standard is adopted to evaluate the platform’s safety performance. Using the aforementioned PSD loading, the dynamic response characteristics of the flexible positioning platform are investigated under this standardized random vibration environment.

Due to the significant uncertainty of random response analysis in the time domain, it is often transformed into the frequency domain for processing in practical research, and the system characteristics are obtained by analyzing its Power Spectral Density (PSD) function. According to the GB/T 2423.56-2023/IEC60068-2-64:2019 standard, the data of spectral inflection points in Table A.8—Equipment mounted on fixed-wing aircraft and helicopters, Category 3a, is used as the excitation condition for the Power Spectral Density (PSD). Under random excitation, the vibration loads on the nodes of the flexible positioning platform change randomly over time, presenting a random process. Its statistical characteristics conform to the Gaussian distribution law. Within the range of one standard deviation (±1σ), the probability of the load occurring is 68.27%. Within the range of two standard deviations (±2σ), the probability increases to 95.45%, and within the range of three standard deviations (±3σ), it can cover almost 99.73% of all possibilities.

**Figure 3:**
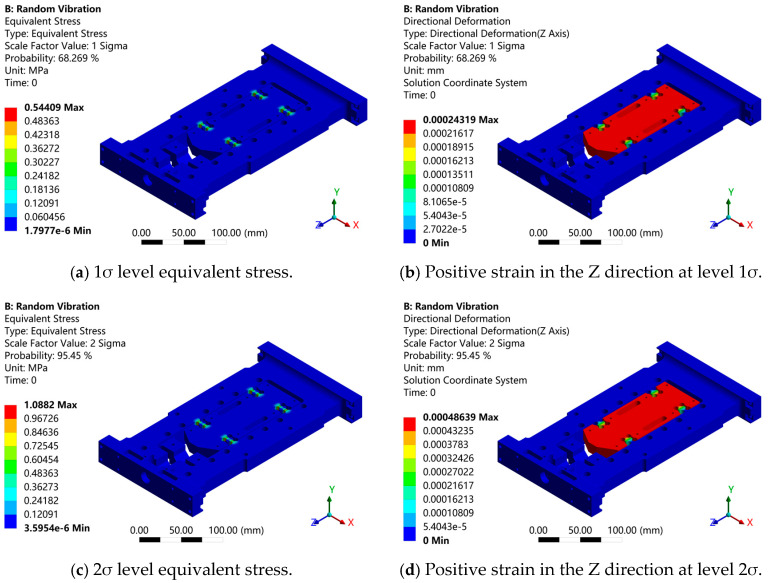

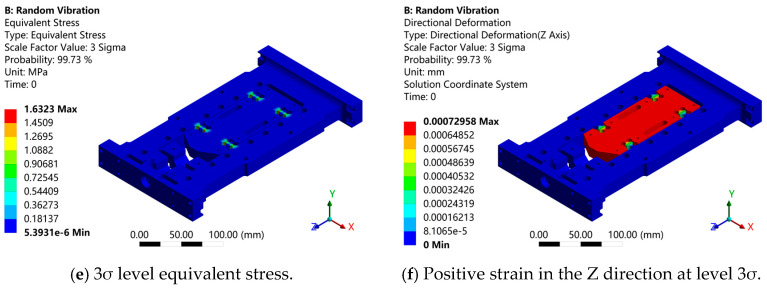
Random vibration response analysis diagram of structure under different σ levels.

**Figure 4:**
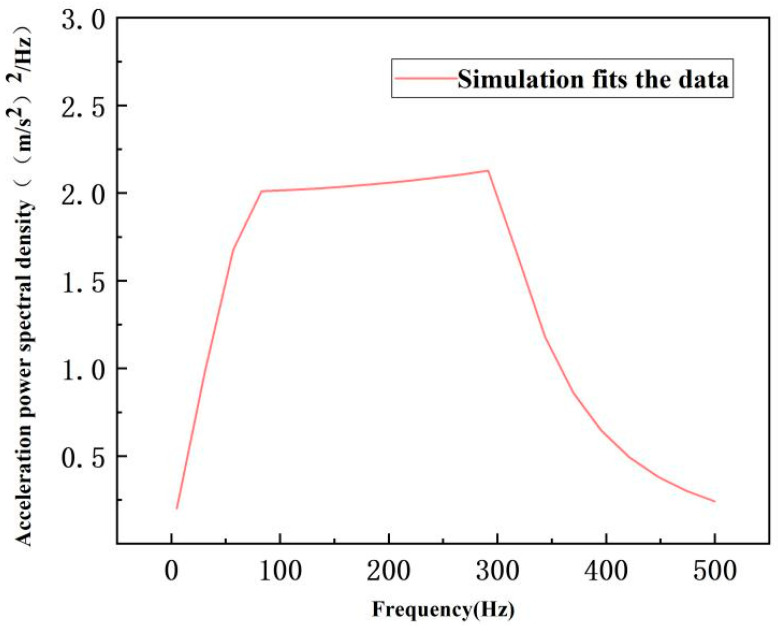
Nodal acceleration power spectral density (PSD) response.

As shown in Figure 3, the equivalent stress distribution of the flexible positioning platform under random acceleration excitation exhibits a consistent pattern across three different Gaussian levels. Stress concentration primarily occurs near the hinge structure, with the equivalent stresses corresponding to the 1σ, 2σ, and 3σ intervals being 0.54409 MPa, 1.0882 MPa, and 1.6323 MPa, respectively. The stress gradually increases from the hinge region toward the micro-motion platform, reaching its maximum at the hinge, indicating that the hinge stiffness plays a critical role in the platform’s vibration resistance. The Z-axis deformation cloud diagrams of the flexible positioning platform under 1σ, 2σ, and 3σ Gaussian distribution intervals show displacement deformations of approximately 0.00024 mm, 0.00049 mm, and 0.00073 mm, respectively. With the increase in the Gaussian interval, the structural deformation exhibits a clear upward trend. The deformation is mainly concentrated in the region connecting the micro-motion platform and the hinge. The deformation observed in the hinge area further underscores the significant impact of hinge stiffness on the vibration resistance of the flexible positioning platform. To evaluate the dynamic response of the structure under random excitation, this study selects a critical node on the micro-motion platform of the flexible positioning platform (located at the force-transmission end of the hinge) as the research subject. The power spectral density (PSD) response curve of this node is depicted in Figure 4. Within the 100–300 Hz frequency range, the response at this node exhibits significant enhancement, with the peak acceleration power spectral density exceeding 2.0 (m/s^2^)^2^/Hz. This amplification phenomenon aligns well with the primary excitation band of the input PSD, confirming this frequency range as the main energy transfer zone of the structure. Beyond 300 Hz, the response PSD demonstrates exponential decay, indicating superior vibration isolation performance of the structure in high-frequency domains.

### 2.4. Random Vibration Analysis Based on Table A.6

**Figure 5:**
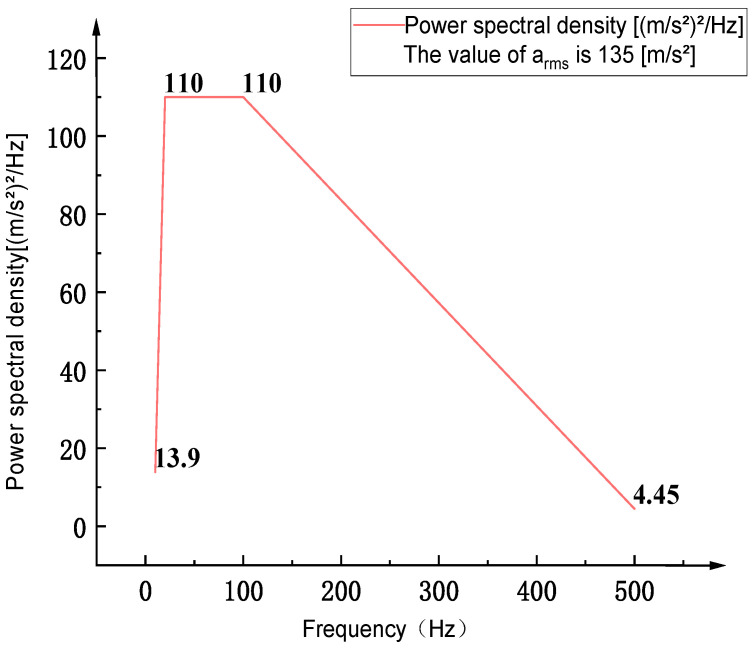
Table A.6 standard acceleration power spectral density curve.

The ultra-high acceleration macro–micro motion platform is driven by dual voice coil motors located at both ends, with a designed acceleration capability of up to 300 m/s^2^. In random vibration analysis, transient acceleration is assumed to follow a Gaussian distribution. According to the 3σ rule, there is a 99.73% probability that the acceleration does not exceed the design limit of the voice coil motors. Based on statistical analysis of vibration amplitude, the instantaneous acceleration under random vibration follows a Gaussian distribution, with the peak acceleration Apeak approximately related to the root mean square (RMS) value by the expression: Apeak ≈ 3 × RMS. In this study, a PSD standard corresponding to an RMS acceleration of approximately 100 m/s^2^ is selected as the research target. The selection of PSD input spectrum is based on Table A.6 of the IEC60068-2-64:2019 standard “Equipment Mounted on Wheeled Vehicles, Class 5b”. The corresponding PSD profile is shown in Figure 5.

**Figure 6:**
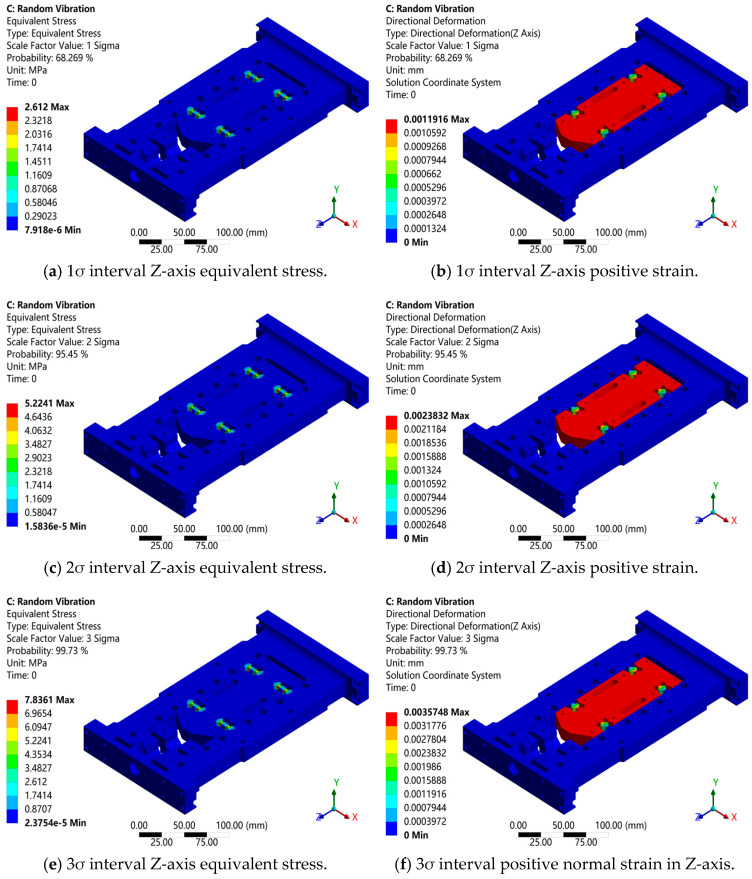
Random vibration response analysis diagram of structures at different o levels.

Random vibration analysis takes the PSD power spectral density data load as the applied excitation condition, and the damping ratio is set to 0.01. The excitation direction is along the z-axis of the global coordinate. The frequency range of the PSD power spectrum is 0–500 Hz. The displacement and stress nephogram of the flexible positioning platform are obtained as shown in Figure 6. The modal superposition method is used to conduct random vibration analysis on the flexible positioning platform. The first six modes are selected as the basis for random vibration analysis. In the analysis of the above PSD excitation standard for the flexible positioning platform, the maximum stress appears at the local hinge. The hinge, as a connecting part, connects the micro-motion platform of the flexible positioning platform. The equivalent stresses at intervals of 1σ, 2σ, and 3σ are 2.612 MPa, 5.2241 MPa, and 7.8361 MPa, respectively. From the perspective of excitation analysis, the overall stress level of the flexible positioning platform is relatively low. The areas with higher stress levels are concentrated at the hinge, indicating that improving the stiffness of this part is the key to optimization and reducing the stress distribution. The stress distribution in the hinge area is relatively uniform, and there is no obvious compressive stress concentration, indicating that the platform structure design has good rationality. The main deformation area of the platform is located at the flexible hinge. The Z-axis deformation nephogram at the Gaussian distribution intervals of 1σ, 2σ, and 3σ has displacement deformations of approximately 0.0012 mm, 0.0024 mm, and 0.0036 mm, respectively. As the interval increases, the greater the excitation deformation and stress become. Compared with the main body of the micro-motion platform, the deformation of this area at the hinge is larger, which indicates that the flexible hinge has a certain elastic deformation ability under random vibration excitation, while other parts of the flexible positioning platform have smaller deformation due to higher structural stiffness.

## 3. Random Vibration Fatigue Analysis of the Flexible Positioning Platform

The ultra-high acceleration macro–micro motion platform is a core piece of equipment in fields such as semiconductor manufacturing, precision machining, and aerospace, owing to its high-precision positioning and rapid dynamic response capabilities. Its operational conditions require the realization of both macro- and micro-scale positioning within extremely short time intervals. In practice, both macro- and micro-motion positioning forces act upon the flexible positioning platform, subjecting it to complex random vibration loads. These stochastic excitations can lead to fatigue damage, ultimately affecting the service life of the platform. Therefore, accurately predicting the fatigue life of the flexible positioning platform under such random loading conditions is crucial for evaluating the performance of key components in ultra-high acceleration macro–micro motion platforms. To enhance the accuracy of fatigue life prediction and structural damage evaluation, a coupled analysis using ANSYS Workbench 2022R1 and nCode DesignLife is performed. First, a harmonic response analysis is conducted with a 2g excitation load applied, and a damping ratio of 0.01 is used. The maximum equivalent stress and strain corresponding to the excitation frequency are obtained as the result data. The random vibration fatigue analysis is then based on these harmonic response results, which are exported and imported into the nCode platform for fatigue life calculation.

**Figure 7:**
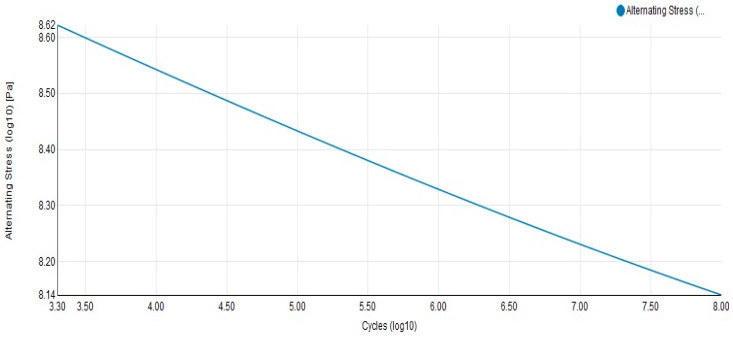
S–N curve of materials.

Through data sharing, material properties and model information are automatically recognized in nCode. The PSD (Power Spectral Density) is selected as the excitation input. In fatigue prediction, the Dirlik method is adopted in conjunction with Miner’s linear damage accumulation rule. The Dirlik method is a frequency-domain analysis technique widely used for evaluating structural fatigue life under random vibration loads, particularly effective in Gaussian narrowband random vibration scenarios. This approach derives strain distribution patterns through statistical characteristics of stress responses, enabling efficient prediction without time-domain reconstruction and utilizing power spectral density data. When applied to non-Gaussian vibrations, the method requires signal decomposition or statistical correction to enhance its applicability. Two acceleration PSD standards mentioned earlier are used as excitation conditions for fatigue prediction and structural damage assessment. The harmonic response and modal analysis results are shared directly with nCode DesignLife. The S–N curve data of the materials used in the flexible positioning platform are derived from nCode’s built-in material library, which provides a wide range of commonly used engineering materials for direct analysis. For the material used in the flexible positioning platform, the Dirlik method is applied to estimate the stress range distribution based on the PSD function. Combined with the S–N curve and Miner’s rule, the fatigue life prediction is carried out. The S–N curve of 7075 aluminum alloy is shown in Figure 7.

### 3.1. Fatigue Analysis Under Table A.8 Standard Random Vibration

Based on the IEC 60068-2-64:2019 standard, the PSD load spectrum corresponding to Table A.8—Spectral Breakpoints for Equipment Mounted on Fixed-Wing and Rotary-Wing Aircraft (Category 3a)—is selected. Using 2g acceleration as the excitation load, and after importing the harmonic response results and defining the material’s corresponding S–N curve and relevant fatigue parameters, the random vibration fatigue life evaluation of the flexible positioning platform can be performed using nCode DesignLife. This analysis yields the corresponding fatigue damage distribution and life prediction results.

**Figure 8:**
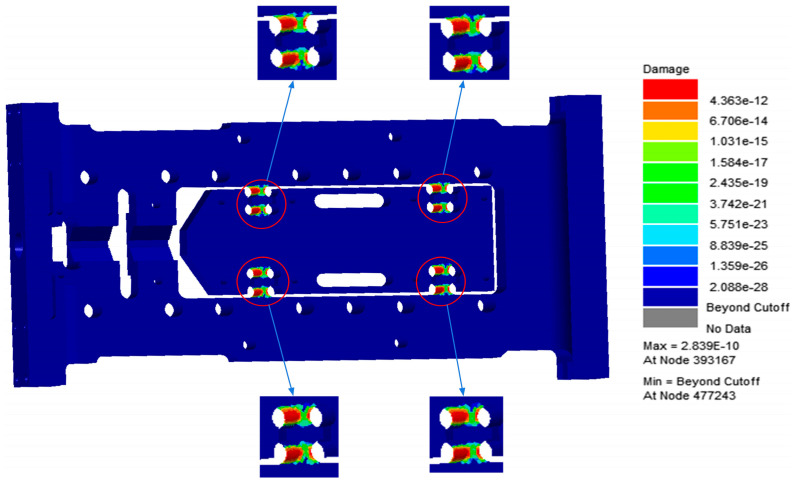
Damage cloud map of flexible positioning platform.

**Figure 9:**
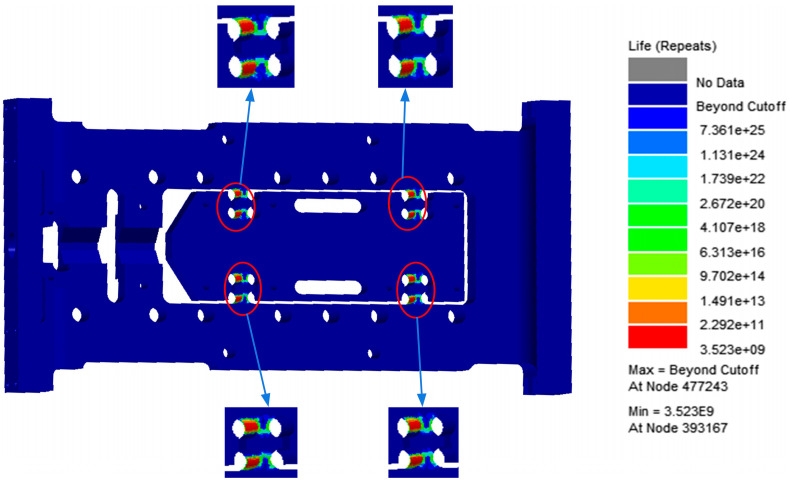
Life cloud map of flexible positioning platform.

As shown in Figure 8 and Figure 9, the fatigue damage contour and fatigue life contour of the flexible positioning platform under random vibration loading are presented, respectively. The analysis results indicate that the shortest fatigue life occurs in the flexible hinge region, identifying this area as the fatigue-prone zone of the platform. Under the excitation of the selected PSD (Power Spectral Density) standard, the overall fatigue damage level is relatively low, with most regions showing a stable trend, demonstrating good resistance to random vibration fatigue and strong fatigue durability. A noticeable concentration of fatigue damage appears at the fillet region of the flexible hinge. The maximum fatigue damage reaches 4.363 × 10^−^^12^, and the minimum fatigue life is 3.523 × 10^9^ cycles. From the fatigue damage and life analysis figures, it can be concluded that the structure of the flexible positioning platform meets the design requirements under this PSD standard. This is further confirmed by the random vibration performance test described below, in which the platform was subjected to a 1 h endurance test based on the Table A.8 standard data, with no structural failure occurring. These results provide a solid foundation for assessing the safety of the platform under higher-energy PSD excitation spectra.

### 3.2. Fatigue Analysis Under Table A.6 Standard Random Vibration

To evaluate the structural durability of the flexible positioning platform under random vibration conditions, the excitation data from Table A.6 of the IEC 60068-2-64:2019 standard was selected. This loading spectrum corresponds to the operating environment of the voice coil motor. The finite element simulation results were imported into nCode DesignLife, where the relevant power spectral density (PSD) load was applied to carry out fatigue analysis under random vibration conditions.

**Figure 10:**
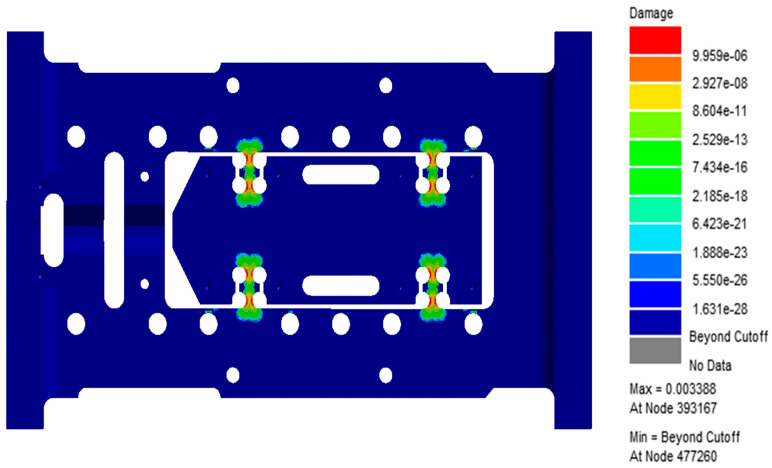
Damage cloud chart of flexible positioning platform.

**Figure 11:**
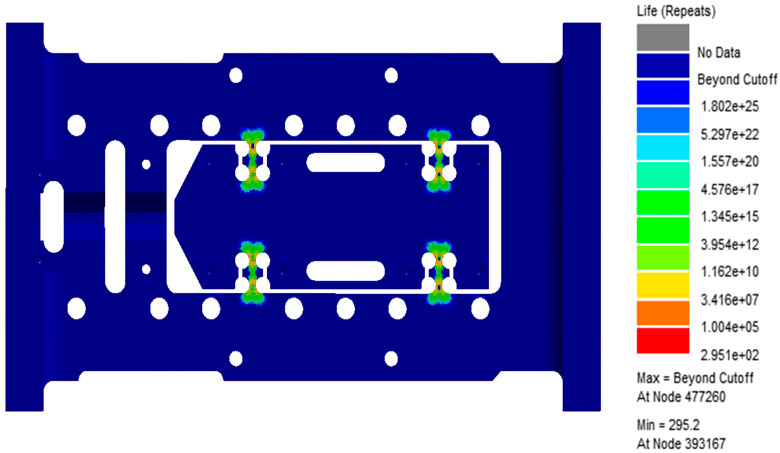
Life cloud chart of flexible positioning platform.

Figure 10 and Figure 11 display the resulting contour plots of fatigue damage and predicted life. From the analysis, it is clear that the flexible hinge region experiences the most severe fatigue damage, with the maximum damage value reaching 9.96 × 10^−6^. The same region also corresponds to the lowest fatigue life, estimated at only 295.2 cycles, suggesting it is the primary weak spot in the current design. Further detailed examination of the fatigue response shows that all of the 20 nodes with the shortest predicted life, as listed in Table 2, are located in the hinge area. The lowest among them, node 39,3167, shows a life of just 295.2 cycles and a damage value of 0.003388. These results point to significant stress concentration in this region under the specified loading, making it a key target for structural improvement. In summary, the findings highlight the need to optimize the hinge design to improve fatigue life, especially when subjected to the vibration conditions described in Table A.6. This section will serve as a baseline for further optimization efforts discussed in the following chapters.

## 4. Optimization Design of the Flexible Positioning Platform

### 4.1. Design Point Selection

**Figure 12:**
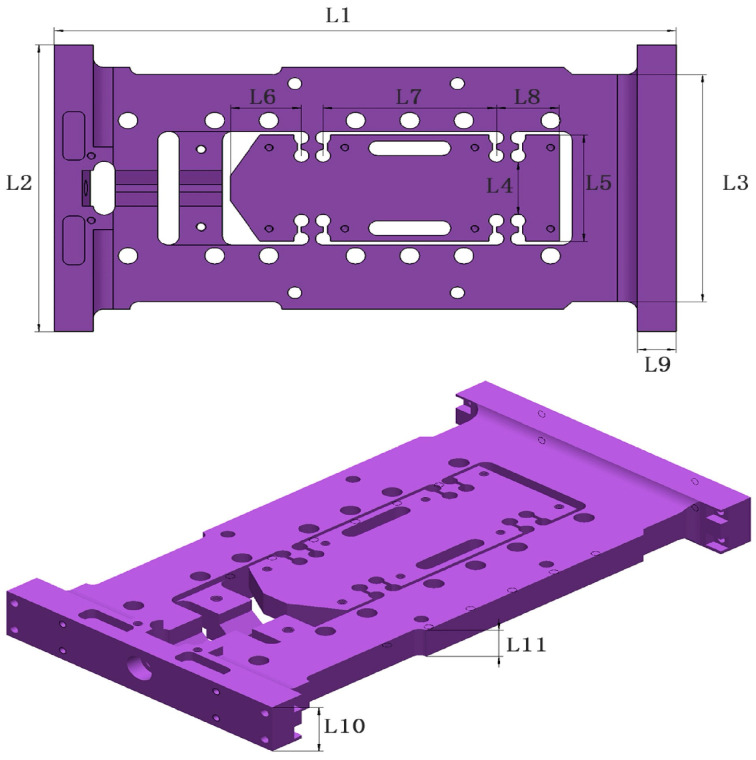
Three-dimensional model of flexible positioning platform and key dimensions of the model.

The flexible positioning platform plays a key role in the ultra-high-speed acceleration macro–micro motion platform, simultaneously performing both macro and micro positioning functions to achieve precise collaborative motion. When the ultra-high-speed macro-micro motion platform achieves ultra-precision positioning, the flexible positioning platform, as shown in Figure 12, plays a crucial role. Table 3 presents the dimensional parameters of the flexible positioning platform.

The hinge area and micro-motion platform area of the flexible positioning platform were optimized to improve the structural performance and accurate positioning ability. Through modal analysis and random vibration analysis, the mass, natural frequency, Z-axial displacement deformation, and maximum equivalent stress of the flexible positioning platform are obtained. The first-order confinement mode has the lowest frequency and is prone to resonance, and large deformation may lead to positioning deviation and structural fatigue damage. The stability and positioning accuracy of the platform under dynamic load conditions are improved by taking the natural frequency of the first-order confined mode, Z-axial displacement, maximum equivalent stress, structural quality, and minimum fatigue life as the optimization goals. The natural frequency, mass, and equivalent stress are selected as optimization objectives to evaluate the resonance characteristics, lightweight performance, and critical stress region hinges, respectively, of the compliant positioning platform, so as to meet the needs of high-precision positioning.

Design variables: X1; X2; X3; X4; X5……Xn.
Objective: minσ(x), maxδ(x), minq(x), minp(x).
Among them: δ(x) is mass, δ(x) is frequency, q(x) is amplitude and p(x) is Equivalent stress.
Design variables:14 mm ≤ X1 = U ≤ 15.5 mm; 2 mm ≤ X2 = V ≤ 6 mm; 7 mm ≤ X3 = L ≤ 10 mm; 12 mm ≤ X4 = W ≤ 15 mm.

### 4.2. Response Surface Optimization Design

This study focuses on optimizing several key dimensions of the flexible positioning platform. Each of the four selected optimization variables corresponds to a critical geometric feature with direct mechanical implications:

U: the horizontal dimension of the top inclination of the micro-motion platform, which governs the positioning of the flexure structure along the X-axis. This parameter affects the local load path and influences the bending resistance and stiffness distribution of the upper segment.

V: the horizontal spacing between the arc centers of the flexure hinge, which defines the effective arm length of the compliant mechanism. This spacing significantly impacts the platform’s flexibility, compliance, and first-order natural frequency.

L: the width of the central long groove, which primarily regulates local flexibility and stress distribution. A wider groove increases deformation capacity but may lead to reduced structural integrity under certain loading conditions.

W: the thickness of the micro-motion platform, which directly affects the bending stiffness, inertia, and mass. While increasing W enhances rigidity and dynamic performance, it also increases the system’s weight and material cost.

These variables were selected based on structural mechanics principles and further evaluated through sensitivity and correlation analysis to ensure their impact on performance metrics such as natural frequency, mass, equivalent stress, and Z-axis deformation.

**Figure 13:**
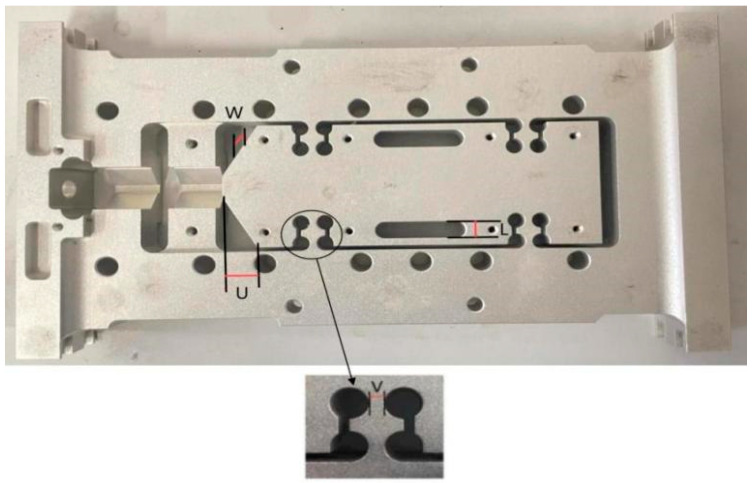
Optimized size diagram of flexible positioning platform.

The structural parameters of the micro-motion platform include the top inclination (U), the arc spacing of the hinge (V), the width of the middle groove (L), and the platform’s thickness (W). The primary objective of optimization is to raise the first-order natural frequency, minimize the peak of modal vibrations, and enhance overall performance while keeping the mass low. To achieve this, a mathematical model of the platform is built. Using the central composite design (CCD) method, a set of design points is generated, followed by the development of a response surface model. Figure 13 illustrates the optimized configuration of the flexible positioning platform. Table 4 presents the corresponding relationship between the design parameters and the optimization objectives, including data from the first 20 design points.

**Figure 14:**
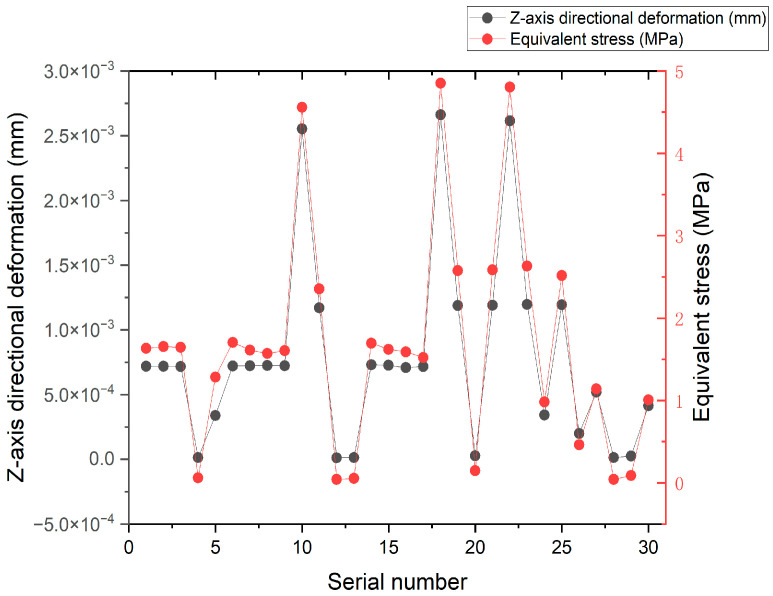
Variation curves of the sample point with Z-axis directional deformation and equivalent stress.

**Figure 15:**
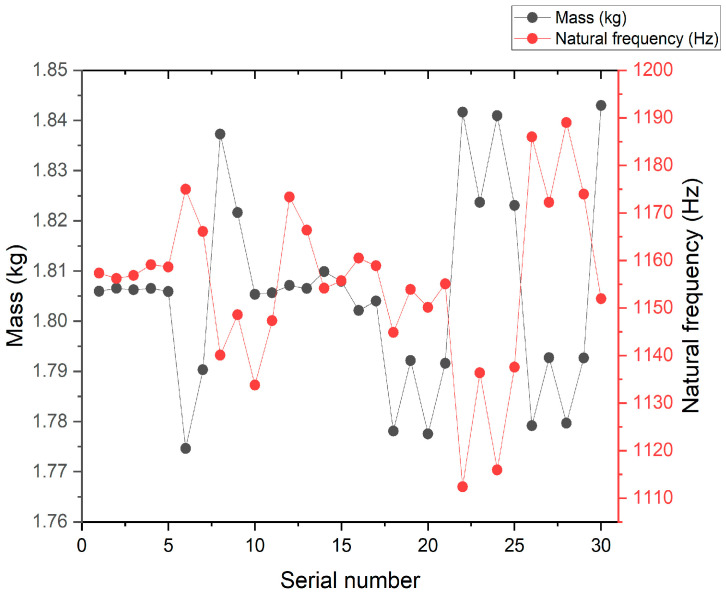
Variation curves of the sample point with mass and natural frequency.

**Figure 16:**
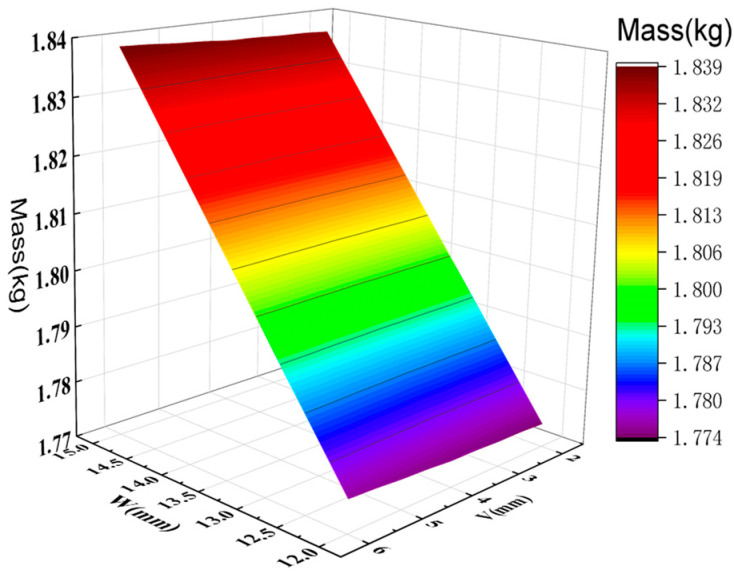
Surface chart of quality and optimization variables.

**Figure 17:**
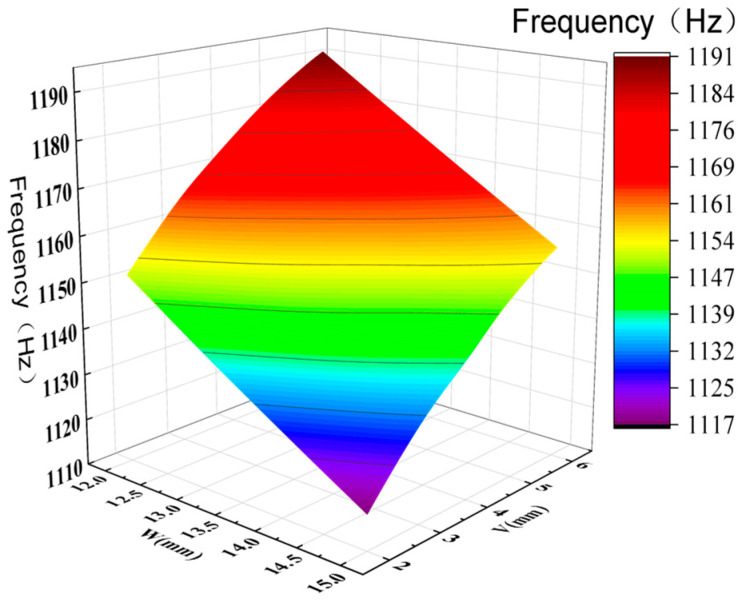
Surface plot of natural frequency and optimization variables.

**Figure 18:**
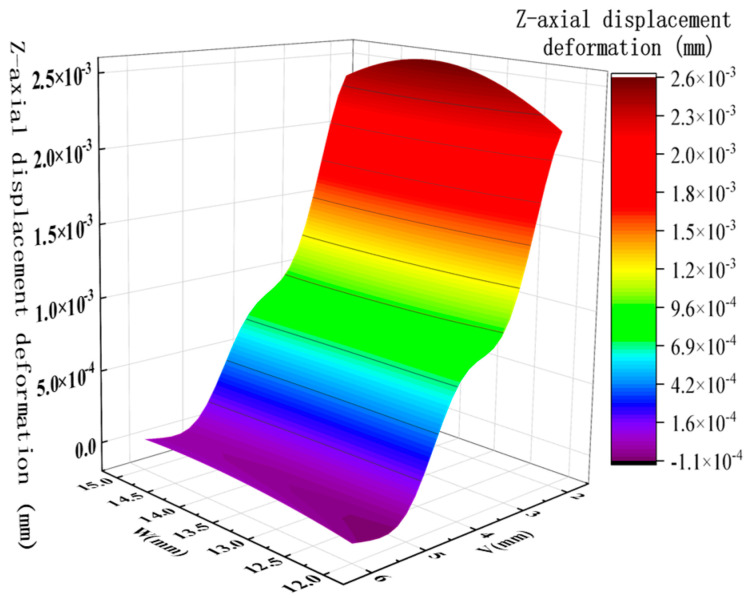
Z-axis displacement deformation and optimization variable surface plot.

**Figure 19:**
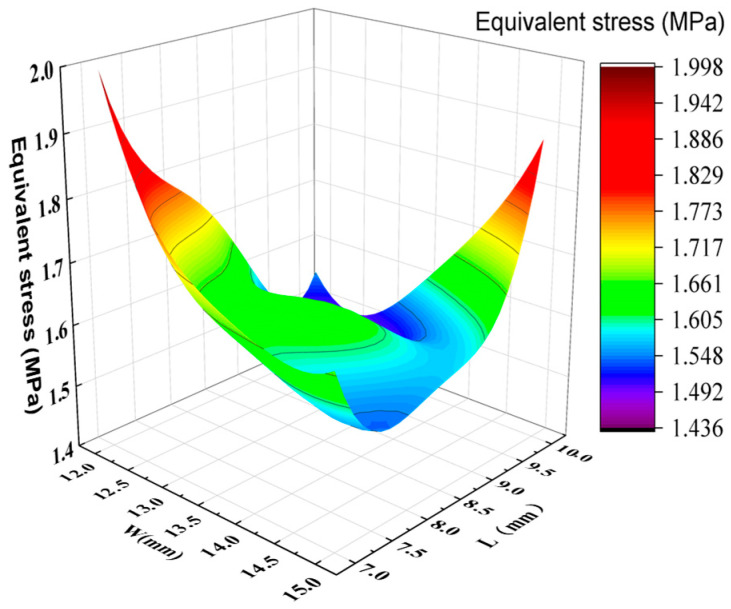
Equivalent stress and surface plot of optimization variables.

By analyzing the variation curves of the sample points, the trends in mass, natural frequency, equivalent stress, and Z-axis deformation were evaluated. As shown in Figure 14 and Figure 15, fluctuations in mass significantly affect the natural frequency, indicating a sensitive coupling relationship between the two. This suggests that a trade-off between mass and frequency is necessary in the optimization process to enhance structural performance. Additionally, the Z-axis deformation and equivalent stress exhibit similar trends, revealing a strong correlation—regions experiencing higher stress often correspond to greater deformation. As shown in Figure 16, the mass increases with the increase in the dimensions W and V, ranging from 1.774 kg to 1.839 kg. As shown in Figure 17, the natural frequency increases with the increase in the dimensions W and V, ranging from 1117 Hz to 1191 Hz. Among them, V has a more significant impact on frequency improvement. As can be seen from Figure 18, the Z-axis displacement deformation decreases with the increase in dimension V, and the maximum deformation occurs at a smaller value of V. As shown in Figure 19, the equivalent stress decreases with the increase in dimension W and increases with the increase in dimension L. To further investigate the influence and interaction of the variables, sensitivity and correlation analyses were conducted using the response surface model.

**Figure 20:**
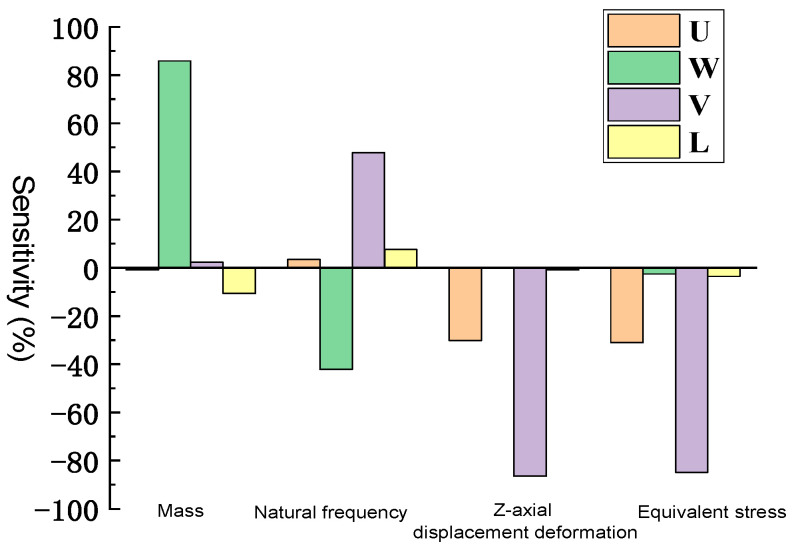
Response surface optimization sensitivity diagram.

As shown in Figure 20, the sensitivity analysis reveals that:

Natural frequency shows a strong positive correlation with V and a moderate negative correlation with W, indicating that increasing the hinge spacing improves dynamic stiffness, while excessive thickness may reduce it due to inertial effects.

Mass is predominantly affected by W and V, both showing positive correlations. W has the greatest contribution to the mass increase.

Z-axis deformation and equivalent stress show a strong negative correlation with U and V, suggesting that appropriate increases in these variables help suppress deflection and stress accumulation.

Moreover, second-order interaction terms in the response surface indicate that:

The combined increase in V and W enhances natural frequency but at the expense of greater mass, revealing a trade-off between stiffness and weight.

Interactions between U and V significantly affect Z-axis deformation, where high U and V jointly reduce deformation.

These findings confirm that variable interactions are non-negligible and should be considered during multi-objective optimization to balance dynamic performance and lightweight requirements.

A positive value on the Y-axis indicates a positive correlation, and a negative value indicates a negative correlation. In the natural frequency column, V shows a positive correlation with the natural frequency, while W shows a mainly negative correlation. The dimensions W and V have a greater influence. W and V show a significant positive correlation with mass. Dimension W is the main factor affecting mass. In the Z-axis directional deformation and equivalent stress, both U and V show a strong negative correlation relationship, indicating that increasing U and V can effectively suppress deformation.

**Figure 21:**
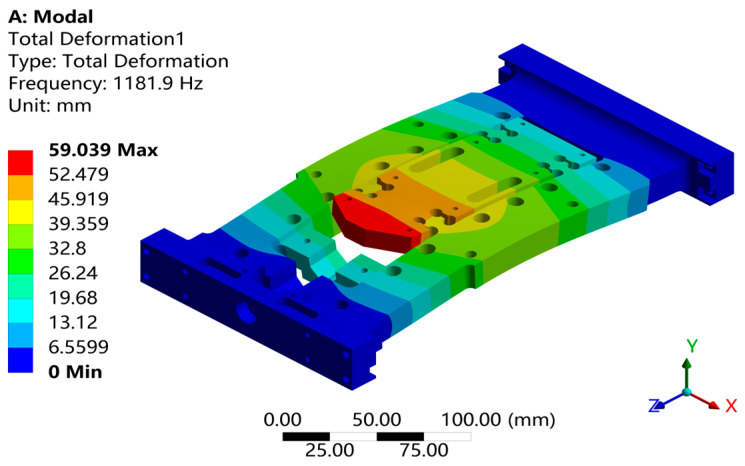
Optimized first-order constrained mode.

**Figure 22:**
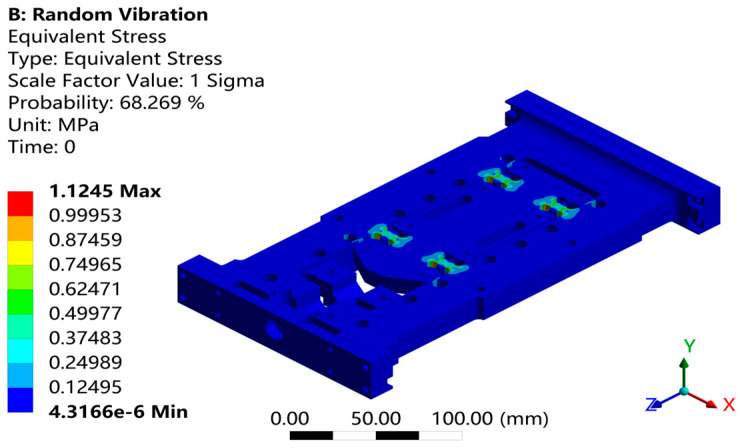
Random vibration equivalent stress under 1σ intervals.

**Figure 23:**
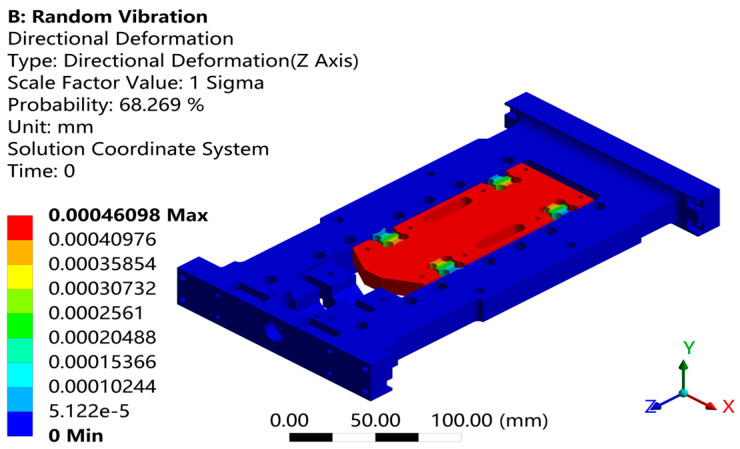
Random vibration displacement nephogram in axial 1σ interval.

**Figure 24:**
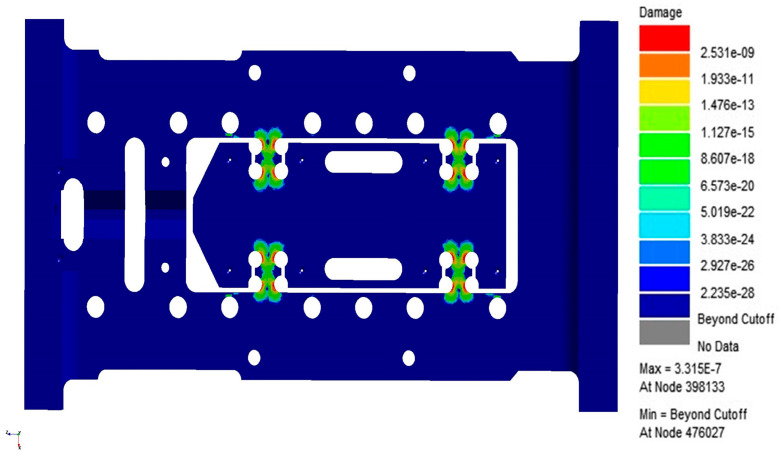
Fatigue damage nephogram optimized based on Table A.6 standard.

**Figure 25:**
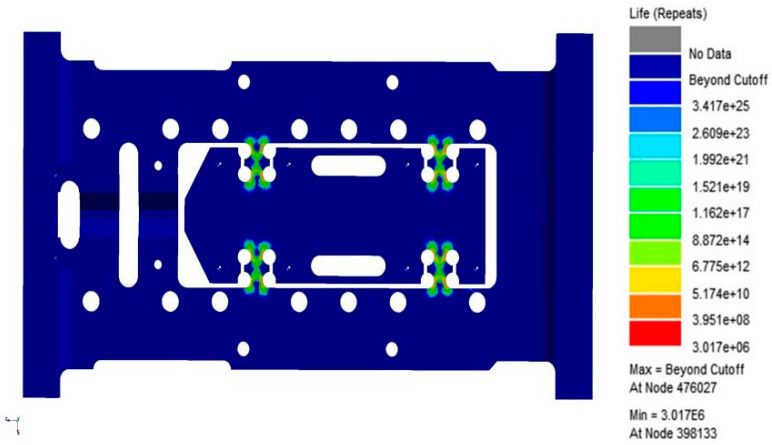
Fatigue life cloud chart optimized based on Table A.6 standard.

Through response surface optimization, the recommended sizes are as follows: the optimized value of “U” is 14.003 mm, “W” is 12.188 mm, “V” is 5.5561 mm, and “L” is 8.9204 mm. After adjusting the structure to these optimized sizes, verification was carried out. As shown in Figure 21, the first-order constrained natural frequency increased from 1137.5 Hz to 1181.9 Hz, with a growth rate of 3.9%.As shown in Figure 22, while the equivalent stress decreased from 2.612 MPa to 1.1245 mm, for a reduction of 56.9%. As shown in Figure 22. As shown in Figure 23, the Z-axis directional deformation significantly decreased from 0.0011916 mm to 0.00046098 mm, a reduction of 61.3%. At the same time, the mass decreased from 1.8175 kg to 1.7785 kg, a decrease of 2.1%. These results show that the optimized design effectively improves the dynamic performance and vibration resistance of the flexible positioning production platform, reduces the vibration response, and achieves the goal of lightweight design. As shown in Figure 24 and Figure 25, the maximum fatigue damage is 2.531 × 10^−9^, and the fatigue life reaches 3.017 × 10^6^. The fatigue life meets the expectation. The optimized structure effectively improves the dynamic stability and vibration resistance of the flexible positioning platform. The comparison table before and after the optimization of the flexible positioning platform is shown in Table 5. The improved performance before and after optimization provides a certain value reference for the optimization of such hinges.

## 5. Flexible Positioning Platform Test

To verify the dynamic performance of the flexible positioning platform structure under actual working conditions, this paper conducts modal tests and random vibration tests. A modal test system is built by LabVIEW equipment to obtain the natural frequency and frequency response characteristics of the structure. At the same time, according to the standard IEC 60068-2-64, a random vibration test is carried out by using an electric vibration table to analyze the response characteristics of the platform under complex vibration environments. The two test methods complement each other. The former is used to identify the modal characteristics of the structure, and the latter is used to evaluate its anti-vibration stability and reliability, aiming to provide experimental support for structural design optimization and simulation model verification.

### 5.1. Modal Test of Flexible Positioning Platform by LabVIEW Method

**Figure 26:**
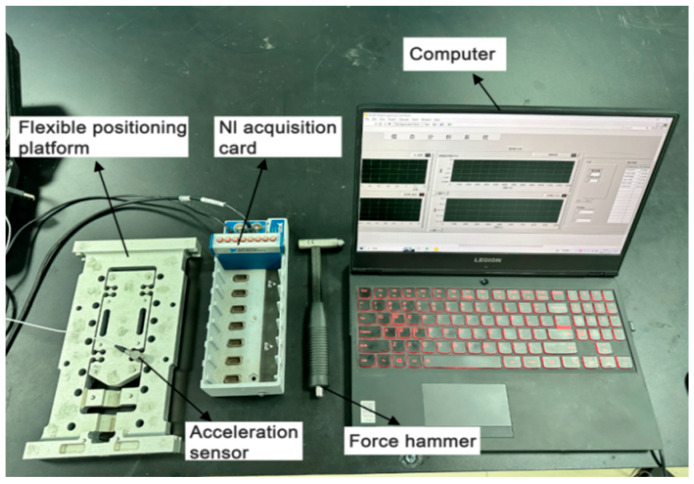
LabVIEW experimental modal testing.

In this experiment, a modal test platform is built by using tools such as the NI data acquisition card, a force hammer, and an acceleration sensor. The models and functions of the instruments and equipment are shown in Table 6. During the experiment, the flexible positioning platform is placed in a suspended state. The predetermined position is struck by a force hammer. At the same time, the measuring point is placed on the micro-motion platform, and an acceleration sensor is pasted. The force signal and acceleration signal generated by the strike are collected by the NI acquisition card and transmitted to the computer. The LabVIEW test program is used for data acquisition and processing. The analyzed resonance frequency is the natural frequency. The test platform is shown in Figure 26.

In this paper, the modal test of the flexible positioning platform is carried out by using the single-point excitation method of the force hammer. Due to the advantages of simple structure, easy operation, and reliable test accuracy, the hammering method has become a widely used test method in modal testing. A total of 11 knocking points is set in the test, which are distributed around and in the central area of the platform. The measuring points are arranged on the micro-motion platform, and the response data is collected by knocking 11 times in turn. In order to avoid the constraint effect on the structure caused by direct fixation, the suspension method is used in the test to approximately realize the free boundary condition and measure the more accurate natural characteristics of the flexible positioning platform. Given that this platform is applied in the field of ultra-precision positioning, if it is directly fixed on the macro–micro motion platform and subjected to impact load, it may damage the core components and then affect its subsequent performance.

**Figure 27:**
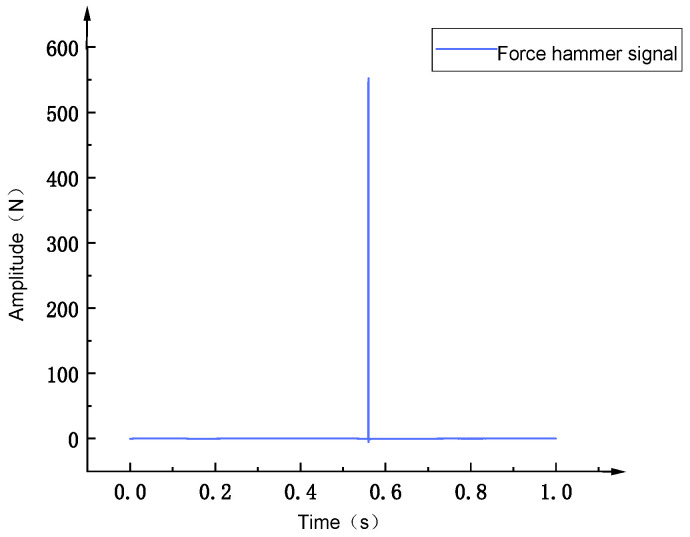
Force hammer signal.

**Figure 28:**
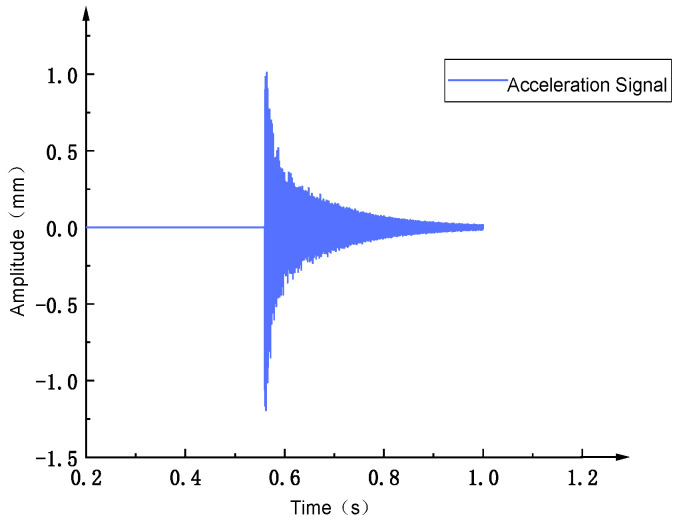
Acceleration signal.

**Figure 29:**
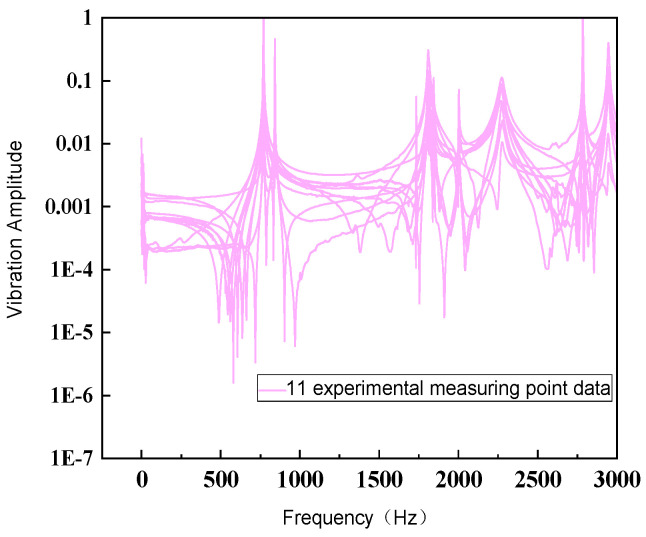
Eleven test point data.

**Figure 30:**
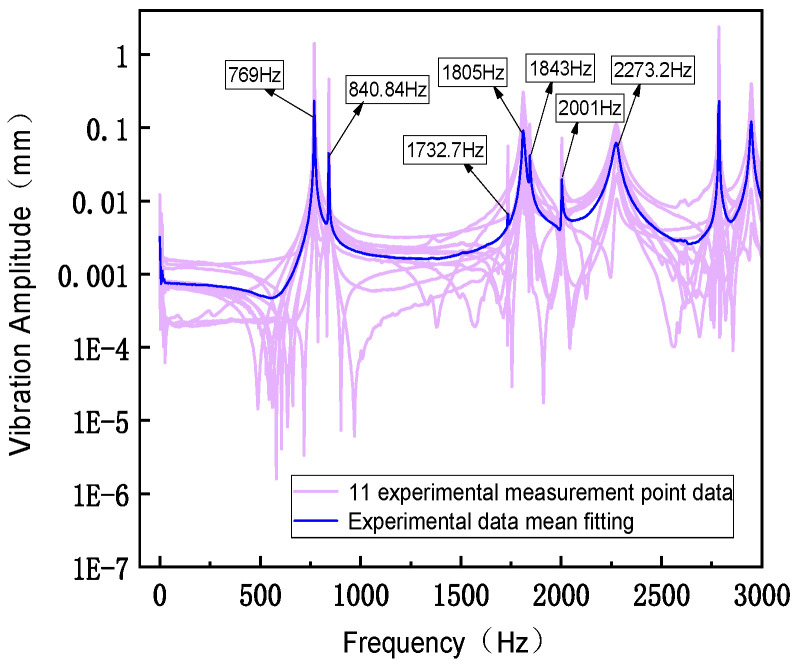
Fitting of test data.

When conducting modal tests in the LabVIEW environment, Figure 27 shows the force hammer signal, which appears as an instantaneous impact force. At a specific time point, it presents a high-amplitude pulse and is almost zero at other times. This force hammer signal is used to excite the natural modes of the structure. Figure 28 shows the corresponding acceleration signal, which appears as a free vibration decay signal of the structure after the impact occurs. Its amplitude decreases with time, reflecting the dynamic characteristics of the system. By analyzing these signals, the modal natural frequency parameters of the structure can be extracted. Figure 29 shows the frequency response function data measured in the modal test, including the response results of a total of 11 test points. The horizontal coordinate is frequency, and the vertical coordinate is amplitude, using logarithmic coordinate scales. Multiple peaks can be observed in the figure, corresponding to the natural frequencies of the structure. The data distribution of different test points indicates the difference and variation in the excitation response of the structure at different positions. As shown in Figure 30, the frequency response function test data of 11 test points of the flexible positioning platform and its average amplitude fitting curve can be seen. The frequency range is from 0 to 3000 Hz. By using the Y-axis average method for fitting, the fluctuation of the data of a single 11 knocking point can be effectively reduced, and the resonance characteristics of the entire platform can be clearly presented. According to the fitted curve, there are a total of six obvious resonance peaks and a weaker peak in the structure. The sequence of peaks before and after corresponds to 769 Hz, 840.84 Hz, 1732.7 Hz, 1809 Hz, 1841 Hz, 2001 Hz, and 2273.21 Hz. The fitted data not only smooths the discrete fluctuations in the test curve but also more accurately reveals the resonance characteristics of each mode, providing analysis for further modal parameter extraction and finding resonance points.

The comparison of the modal test and finite element simulation results is shown in Table 7. Generally speaking, the finite element analysis of the first six modes is in good agreement with the test results. The maximum error does not exceed 7%, reflecting the high simulation accuracy of the finite element method calculation. The frequency error of the first mode is the lowest (only 0.23%), indicating that the finite element model has high prediction accuracy in the low-frequency region and the model construction and parameter selection are reasonable. The errors of the second, fourth, and fifth modes are 1.15%, 3.45%, and 2.89%, respectively, all within a reasonable error range, indicating that the dynamic characteristics of the simulation model and the actual structure are in good consistency. The modal error of the third order is slightly larger at 6.68%, and the natural frequency error of the sixth-order mode is 4.2%, which may be related to the test measurement points on the micro-motion platform. According to the above analysis, the finite element simulation and test results show good consistency as a whole, verifying the effectiveness and reliability of finite element modeling.

### 5.2. Random Vibration Performance Test

**Figure 31:**
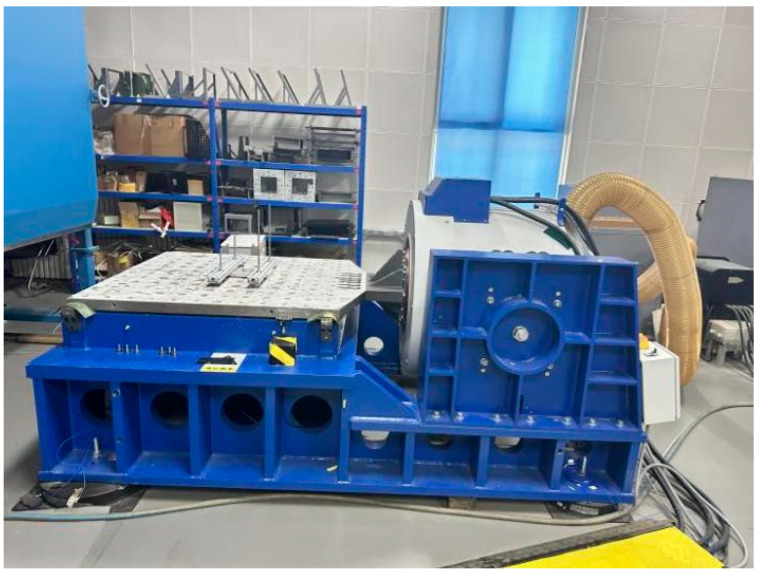
Random vibration performance test.

In order to study the dynamic response characteristics of structures under random vibration environments, based on the IEC 60068-2-64 standard, this study conducts random vibration tests using an electric vibration table based on the data in Table A.8. The test system is mainly composed of a vibration table, a power amplifier, a control system, an acceleration sensor, and a data acquisition and analysis system. The test and measurement equipment are shown in Figure 31. The equipment used for random vibration testing is shown in Table 8. The vibration table is used to provide vibration input that meets the power spectral density (PSD) setting. The power amplifier ensures that the excitation signal strength meets the test requirements. The acceleration sensor monitors the dynamic response of the product in real time and records the vibration characteristics through the data acquisition system. In the test, a closed-loop control system is used to keep the error between the PSD output by the vibration table and the target PSD within the accuracy range to ensure the stability of the test.

**Figure 32:**
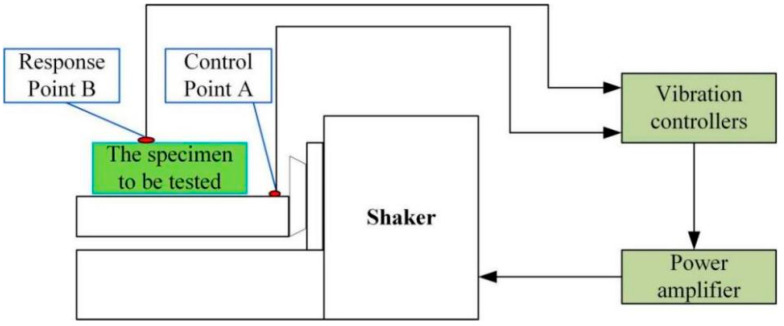
Schematic diagram of random vibration performance test.

**Figure 33:**
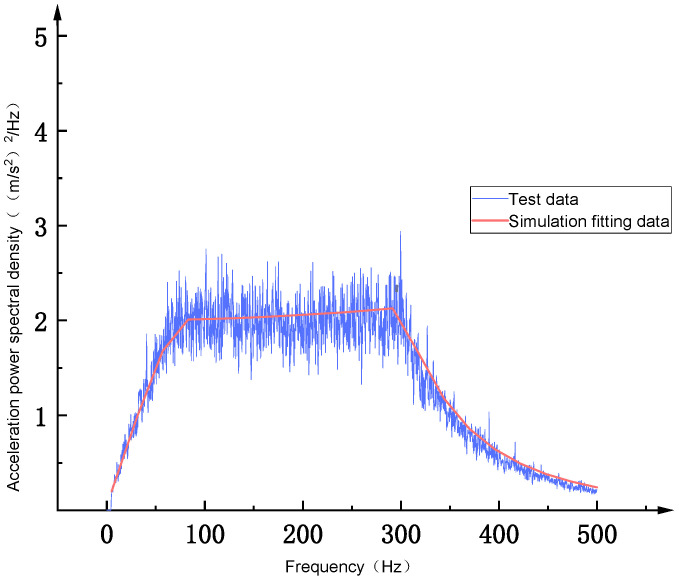
Comparison of test data and simulation data for random vibration test.

As shown in Figure 32, a schematic diagram of the brief principle of a random vibration test system is presented. The test system is mainly composed of a vibration table, a power amplifier, a vibration controller, and a tested piece. The vibration control point a and the response measurement point B are placed at the installation location and on the flexible positioning platform, respectively. During random vibration testing, the vibration force controller outputs a random vibration control signal. After being amplified by the power amplifier, it drives the vibration table to generate random vibration excitation and simulate the excitation on the tested piece. The response signal of the flexible positioning platform structure is collected from the acceleration sensor and fed back to the vibration controller to form a closed-loop control system to realize the real-time adjustment and control of the output signal of the vibration table, thereby ensuring the accuracy and stability of the random vibration test. Combined with ANSYS Workbench for finite element random vibration analysis, the modal superposition method is used for analysis. The structural response PSD at the same position is analyzed and calculated. The simulation results have a high degree of fitting with the test data, verifying the accuracy of the simulation calculation, as shown in Figure 33. The analysis shows that as the test frequency increases, the acceleration power spectral density measured by the acceleration sensor remains stable, ensuring the safety of the product and providing support for research on higher random excitation load standards. This research provides a methodology combining experiment and simulation for predicting the dynamic behavior of complex structures in a random vibration environment and provides a reliable basis for the optimization research of structures.

## 6. Conclusions

This study presents a systematic method for evaluating and optimizing the dynamic performance of a flexible positioning platform under ultra-high acceleration conditions. The key conclusions and design implications are as follows:

- Test and simulation collaborative verification method. For the first time, the hammering modal test based on LabVIEW is combined with the random vibration test to comprehensively evaluate the modal characteristics and dynamic response behavior of the platform, and the results are compared with the finite element simulation results to verify the accuracy and feasibility of the simulation model.
- Finite element modal analysis revealed that the first six natural frequencies of the platform are well distributed, and the stress concentration areas are mainly located at the flexure hinge regions, indicating structural sensitivity in these areas.
- By applying typical PSD load profiles specified in Tables A.6 and A.8 of the IEC 60068-2-64 standard, frequency-domain responses under different random vibration intensities were obtained. The simulation results reflect realistic environmental conditions and demonstrate that the platform experiences greater deformation under higher-intensity spectra.
- Generalizable framework for compliant mechanism evaluation: The methodology combining IEC-standard PSD excitation, ANSYS Workbench modal superposition, and nCode fatigue prediction provides a scalable template for dynamic evaluation of precision flexure structures in aerospace, micro-positioning, and semiconductor applications.

## Figures and Tables

**Table 1:** Natural frequencies of free mode and constrained mode of the flexible positioning platform.

Order	First Stage	Second Stage	Third Stage	Fourth Stage	Fifth Stage	Sixth Stage
Free mode (Hz)	767.2	850.53	1848.4	1871.4	1894.2	2085
Constrained mode (Hz)	1137.5	1549	1712	2202.6	2379.2	2565.5

**Table 2:** Life node data of flexible positioning platform.

Serial Number	Node Number	Damage	Service Life (Times)
1	393,167	0.003388	295.2
2	392,294	0.003292	303.8
3	397,574	0.003273	305.5
4	395,160	0.003161	316.4
5	390,445	0.003161	316.4
6	392,594	0.003156	316.9
7	52,321	0.003113	321.2
8	396,524	0.003112	321.4
9	396,845	0.00304	328.9
10	397,572	0.003024	330.7
11	397,149	0.00295	339
12	393,002	0.002896	345.3
13	401,216	0.002893	345.6
14	400,545	0.002881	347.1
15	398,688	0.002851	350.7
16	50,719	0.002851	350.7
17	393,302	0.00283	353.3
18	388,576	0.002812	355.6
19	388,779	0.002807	356.2
20	388,817	0.002797	357.5

**Table 3:** Key dimension values of flexible positioning platform.

Name	L1	L2	L3	L4	L5	L6
Value (mm)	287	159	126	29	59	33
Name	L7	L8	L9	L10	L11	
Value (mm)	80	29	18	30	18	

**Table 4:** The first 20 generated sample points before optimization design.

Serial Number	U	W	V	L	Mass (kg)	Natural Frequency (Hz)	Z-Axis Directional Deformation (mm)	Equivalent Stress (MPa)
1	14.75	13.5	4	8.5	1.806	1157.3	0.00072	1.6323
2	14	13.5	4	8.5	1.8065	1156.2	0.00072	1.6537
3	14.375	13.5	4	8.5	1.8063	1156.8	0.00072	1.6454
4	15.5	13.5	4	8.5	1.8065	1159.1	0.00001	0.0605
5	15.125	13.5	4	8.5	1.8059	1158.6	0.00034	1.2853
6	14.75	12	4	8.5	1.7746	1174.9	0.00072	1.7052
7	14.75	12.75	4	8.5	1.7903	1166.1	0.00072	1.6118
8	14.75	15	4	8.5	1.8373	1140.1	0.00072	1.5696
9	14.75	14.25	4	8.5	1.8216	1148.5	0.00072	1.606
10	14.75	13.5	2	8.5	1.8054	1133.8	0.00255	4.5584
11	14.75	13.5	3	8.5	1.8057	1147.3	0.00117	2.3537
12	14.75	13.5	6	8.5	1.8071	1173.3	0.00001	0.0414
13	14.75	13.5	5	8.5	1.8065	1166.4	0.00001	0.0542
14	14.75	13.5	4	7	1.8099	1154.1	0.00073	1.6966
15	14.75	13.5	4	7.75	1.8079	1155.7	0.00073	1.6218
16	14.75	13.5	4	10	1.8021	1160.5	0.00071	1.5902
17	14.75	13.5	4	9.25	1.804	1158.9	0.00072	1.522
18	14	12	2	7	1.7781	1144.8	0.00266	4.8509
19	14.375	12.75	3	7.75	1.7921	1153.9	0.00119	2.5773
20	15.5	12	2	7	1.7775	1150.1	0.00003	0.1488

**Table 5:** Comparison table of results before and after optimization of flexible platform.

Optimization Objective	Before Optimization	After Optimization	Comparison (%)
First-order constraint modal natural frequency (Hz)	1137.5	1181.9	+3.9
First-order constraint modal natural frequency (Hz)	0.0011916	0.00046098	−61.3
Maximum equivalent stress (MPa)	2.612	1.1245	−56.9
Structural mass(kg)	1.8175	1.7785	−2.1
Minimum fatigue life (times)	2.95 × 10^2^	3.02 × 10^6^	+1.02 × 10^4^

**Table 6:** Equipment used in experimental modal analysis.

Name	Model	Purpose
data acquisition card	NI9230	Acquire signal
force hammer	PCB086C03	Excitation and acquisition of signal
accelerometer	PCB352C03	Excitation and acquisition of signal

**Table 7:** Comparison of calculation errors between test model and finite element method.

Order	Test Mode (Hz)	Finite Element Mode (Hz)	Error
Level 1	769	767.2	0.23%
Level 2	840.84	850.53	1.15%
Level 3	1732.7	1848.4	6.68%
Level 4	1809	1871.4	3.45%
Level 5	1841	1894.2	2.89%
Level 6	2001	2085	4.2%

**Table 8:** Testing equipment and functions.

Name	Model	Purpose
Electromagnetic vibration table	-	Provide PSD (power spectral density) vibration excitation
Sensor signal conditioner	PCB piezoelectric element 482C series	Piezoelectric sensor provides power and signal conditioning
Network switch	TP-Link TL-SG1008	Connect and transmit data between controller and computer
Power amplifier	-	Amplify the signal
Acceleration sensor	357B03	Pick up the response signal
Vibration controller	-	Excitation and pick up the signal

## Data Availability

Data available on request due to restrictions on privacy.
